# Development of a Novel Lipid-Based Nanosystem Functionalized with WGA for Enhanced Intracellular Drug Delivery

**DOI:** 10.3390/pharmaceutics14102022

**Published:** 2022-09-23

**Authors:** Gabriela Hädrich, Gustavo Richter Vaz, Juliana Bidone, Virginia Campello Yurgel, Helder Ferreira Teixeira, Alexandre Gonçalves Dal Bó, Luciano da Silva Pinto, Mariana Appel Hort, Daniela Fernandes Ramos, Antonio Sergio Varela Junior, Pedro Eduardo Almeida da Silva, Cristiana Lima Dora

**Affiliations:** 1 Department of Pharmaceutical Technology and Biopharmacy, University of Vienna, Althanstraße 14, 1090 Vienna, Austria; 2 Graduate Program in Health Sciences, Federal University of Rio Grande, Rio Grande 96203-900, Brazil; 3 Center of Chemical, Pharmaceutical and Food Sciences, Federal University of Pelotas, Pelotas 96010-610, Brazil; 4 Graduate Program in Pharmaceutical Sciences, Federal University of Rio Grande do Sul, Porto Alegre 90610-000, Brazil; 5 Graduate Program in Science and Materials Engineering, University of the Extreme South of Santa Catarina, Criciúma 88806-000, Brazil; 6 Graduate Program in Biotechnology, Campus Capão do Leão, Federal University of Pelotas, Pelotas 96010-610, Brazil; 7 Biological Sciences Institute, Federal University of Rio Grande, Rio Grande 96203-900, Brazil

**Keywords:** nanostructured lipid carriers, WGA, intracellular drug delivery

## Abstract

Despite a considerable number of new antibiotics under going clinical trials, treatment of intracellular pathogens still represents a major pharmaceutical challenge. The use of lipid nanocarriers provides several advantages such as protection from compound degradation, increased bioavailability, and controlled and targeted drug release. Wheat germ agglutinin (WGA) is known to have its receptors on the alveolar epithelium and increase phagocytosis. The present study aimed to produce nanostructured lipid carriers with novel glycosylated amphiphilic employed to attach WGA on the surface of the nanocarriers to improve intracellular drug delivery. High-pressure homogenization was employed to prepare the lipid nanocarriers. In vitro, high-content analysis and flow cytometry assay was employed to study the increased uptake by macrophages when the nanocarriers were grafted with WGA. A lipid nanocarrier with surface-functionalized WGA protein (~200 nm, PDI > 0.3) was successfully produced and characterized. The system was loaded with a lipophilic model compound (quercetin; QU), demonstrating the ability to encapsulate a high amount of compound and release it in a controlled manner. The nanocarrier surface functionalization with the WGA protein increased the phagocytosis by macrophages. The system proposed here has characteristics to be further explored to treat intracellular pathogens.

## 1. Introduction

Novel drug delivery systems are designed to deliver the active pharmaceutical ingredient (API) to target sites by avoiding degradation, delivering and maintaining the doses at the tissue of interest consequently improving the therapeutic outcomes, and preventing adverse effects [1]. Among different types of carriers, lipid-based ones have become interesting on account of providing several advantages, such as protection of active compounds, increased drug bioavailability, and controlled drug release. These carriers are colloidal matrix systems or reservoirs, with a size between 10 and 1000 nm, characterized not only by the ability to carry active compounds protecting them from degradation after administration but also by presenting the drugs in a highly dispersed state with a high contact surface for absorption [2]. A lipid carrier can consist of a mixture of solid and liquid lipids, so-called nanostructured lipid carriers (NLC). The NLC system is suitable because it provides the advantages of increasing the entrapment efficiency and extending drug release, appearing to be promising for the encapsulation of lipophilic compounds to be administered by a variety of administration routes. Thus, it is an interesting platform for improving the solubility of poorly water-soluble drugs [3].

The lipid nanocarriers, in addition to transporting drugs, can also be functionalized by components on their surfaces to direct the compounds to the target of interest. This allows the increase of the therapeutic efficiency by prolonging the permanence of the carriers in the absorption site, increasing the bioavailability, or interacting with a specific receptor on the surface of the target cell [4]. An excellent representative of such specific ligands is lectins, which are naturally occurring proteins or glycoproteins capable of binding specifically and reversibly to carbohydrates and glycoconjugates [5,6]. These proteins display receptor-mediated bio-adhesion by recognizing and adhering to glycosylated structures [7,8]. Indeed, a nanotechnology-based lipid carrier with a targeting moiety on the surface represents a novel promising tool.

The recognition of carbohydrates by lectins is the basis for their use in the area of drug targeting. The binding of lectins typically involves the two or three terminal sugar residues of the mammalian glycans including galactose, mannose, *N*-acetyl-neuraminic acid, fucose, or *N*-acetyl-glucosamine [9,10,11]. Among these sugar-binding proteins, the Wheat germ agglutinin (WGA) is known to recognize *N*-acetyl-glucosamine receptors (GlcNAc) in the alveolar epithelium and to increase phagocytosis [12]. Thus, if used to cover nanoparticles, it could increase their adhesion to the pulmonary epithelium, allowing vectorization to alveolar macrophages, a pathway that can be exploited for the enhanced uptake of drug-loaded nanoparticles [12,13,14]. Several nanoparticles were already grafted with WGA on their surface [12,15,16,17,18,19] and, in all cases, the chosen method for surface functionalization is composed of several steps. Here we are using, for the first time, novel surfactants that allow the WGA binding on the surface through click-chemistry in one single step.

Aiming to circumvent the limitations of conventional therapeutic schemes and propose new pharmacological alternatives treatments, in this study, we functionalized with WGA lectin our previously described nanostructured lipid nanocarriers [20] and entrapped them with lipophilic compounds (quercetin, Nile red, and conjugated polymer CNPPV) to better understand how they are released from the nanocarriers and also to analyze the cellular uptake. We hypothesized that nanoparticles functionalized with WGA could improve the intracellular drug payload via lectin receptors expressed on macrophages. 

The treatment of intracellular infections presents several unusual challenges. The antibiotic concentration in those specialized niches is often subtherapeutic, for which high doses of antibiotics must often be used. As a consequence, the regimen is not only costly but may also increase localized or systemic side effects. There is therefore an urgent need for formulations that enable the achievement of therapeutically effective intracellular concentrations of those antibiotics [21]. The idea here is to take advantage of this higher uptake to treat intracellular bacterial pathogens that cause a wide range of diseases and significantly contribute to the morbidity and mortality associated with infectious diseases worldwide. The use of WGA can be promising not only to treat diseases caused by bacterial pathogens but could also be an interesting alternative in the search for drugs for prophylaxis and treatment of viral infections, such as SARS-CoV-2.

Some bacterial pathogens can replicate in macrophages. Paradoxically, the phagocytic cells tailored to kill and digest the ingested material are a satisfying habitat for pathogens such as Listeria, Brucella, Mycobacterium, or Salmonella [21]. As an example of a mechanism, we can cite Mycobacterium tuberculosis due to its capacity to avoid terminal phagolysosome formation and block phagosome maturation [22]. In this context, drug delivery systems using encapsulation nanotechnologies have evolved, and the strategy shown here is to use lipid nanoparticles decorated with WGA to increase the drug cellular intake which could be explored for the treatment of several infective diseases.

## 2. Materials and Methods

Trimyristin (Dynasan 114), was donated by Cremer^®^ (Hamburg, Germany). Egg lecithin E80 was purchased by Lipoid^®^ (Ludwigshafen, Germany). The material used as a conjugated polymer (CN-PPV), Nile red, wheat germ agglutinin conjugated with fluorescein (WGA-FITC), surfactant (PEG-660 stearate), castor oil (CO), Dulbecco’s Modified Eagle’s Medium (DMEM), quercetin (QU), porcine gastric mucin (type II), rat red cell suspension, N-acetylglucosamine, PBS-BSA solution, antibody anti-WGA, anti-rabbit IgG produced in goat, dialysis bag MWCO 10000, and Ortho-Phenylene-Diamine were purchased by Sigma-Aldrich (Saint Louis, MO, EUA). The modified N-Acetyl-b-D-glucosaminyl-PEG900-stearate conjugate (C_18_PEG_900_GlcNAc) was synthesized by Alexandre G. Dal Bó [10,11,23]. WGA lectins were extracted by Luciano Pinto. The other reagents used were of analytical grade.

### 2.1. Development and Characterization of the Lipid Nanocarriers 

The NLC (Table 1) was prepared following a method previously described [20]. Briefly, the oil phase containing trimyristin, castor oil, and egg lecithin E80 was melted at 82 °C, under stirring (1500 rpm) until a homogeneous melt was achieved. A final volume of 10 mL of aqueous phase containing surfactant, a mixture of PEG-660 stearate/C_18_PEG_900_GlcNAc (50:50) at 0.5% (*w*/*v*) and PBS, was heated up until 60 °C and gently poured into the oil phase with a glass bar. After that, the formulation was mixed in a high-performance dispersing instrument (Ultraturrax, IKA^®^) for 2 min at 14,500 rpm. Finally, the obtained emulsion was subjected to 6 cycles of 10,000 psi in the high-pressure homogenizer (C3, Avestin^®^, Ottawa, ON, Canada). The prepared formulations were cooled to room temperature and stored at 4 °C. The NLC containing QU (NLC-QU) as a lipophilic drug model was prepared for evaluation of the drug release behavior from the system and Nile Red or the conjugated polymer CNPPV was entrapped into the system (NLC-CNPPV or NLC-NR) aiming to perform macrophage internalization assays.

The particle size and zeta potential of the nanocarriers were determined by dynamic light scattering and laser-doppler anemometry, respectively, using a Zetasizer Nano Series (Malvern Instruments, Worcestershire, UK) at a scattering angle of 173°. Particle size measurements were performed at 25 °C after appropriate dilution of the samples in NaCl 1 mM. Stokes–Einstein’s equation was used to determine the hydrodynamic radius. For the zeta potential measurements, the samples were placed in the electrophoretic cell with a potential of ±150 mV, and the values were calculated using Smoluchowski’s equation as mean electrophoretic mobility values. All measurements were performed in triplicate and at 25 °C.

### 2.2. Nanocarriers Surface Functionalization

Nanocarriers were produced with 50% of the sugar-modified surfactant, called C_18_PEG_900_GlcNAc, and 50% of the commercial surfactant, PEG-660 stearate. The NLCs with WGA on the surface (NLC-WGA) were obtained by post-insertion. The method consists of the post-preparation incubation of the formulations with dispersions of lectins in PBS pH 7.2 at a concentration of 4 µg/mL. Despite the link being an instant carbohydrate-specific protein interaction, the formulations were subjected to magnetic stirring for 10 min after the addition of WGA [10,11,23]. For uptake studies, commercial lectin conjugated to the fluorophore FITC was used.

### 2.3. Evaluation of the Presence and Activity of Lectins WGA on the Surface of the Nanocarriers

#### 2.3.1. Hemagglutinating Activity (HA)

To evaluate the presence and maintenance of WGA lectin activity in formulations, 100 µL of the samples were subjected to a series of dilutions, and then the diluted samples were incubated with 100 μL of 2% rat red cell suspension. Readings of red blood cells agglutination were visualized after 30 and 60 min.

#### 2.3.2. Enzyme-Linked Immunosorbent Assay

To detect the WGA lectin and verify its functionality after the preparation of the formulation, an ELISA test was performed [24]. Briefly, a 96-well plate was precoated with 0.1% porcine gastric mucin (Type II, Sigma-Aldrich, Saint Louis, MO, USA) overnight. A PBS-BSA solution (0.1%) blocked nonspecific bonds. To perform a calibration curve, protein WGA diluted in PBS (15–1000 ng/mL) and samples (NLC and NLC-WGA) were added to the plate and incubated for 1 h. After washing the plate three times, the primary antibody (anti-WGA 1:2000) was added and incubated for 1 h. Afterward, the PBS was used to wash the plate, and the antibody conjugated with peroxidase (anti-rabbit IgG produced in goat 1:5000) was added. The substrate used for peroxidase activity detection was Ortho-Phenylene-Diamine (OPD). After 30 min, the addition of sulfuric acid (2.5 M) solution stopped the reaction, and the resulting absorbance was read on a spectrophotometer at 490 nm.

### 2.4. Determination of Drug Content and Release Profile

An analytical HPLC method previously developed and validated according to ICH (2005) was used to determine the content and recovery rate of QU from the nanocarriers (NLC-QU) [25,26]. The determination of the drug content was performed on an HPLC (Agilent 1100 series). The detector was set at 369 nm and the experiments were carried out using a reversed-phase Zorbax ODS Agilent Technologies^®^ (Wilmington, NC, USA) C18 column (150 mm × 4.6 mm I.D., with a particle size of 5 μm), maintained at 40 ± 1 °C. The mobile phase consisted of a 1 % phosphoric acid: methanol mixture (45:55 *v*/*v*; pH 2.7) and was eluted isocratically at a flow rate of 1 mL min^–1^.

In vitro release studies were conducted according to the methodology described above [27] to establish the percentage of QU released over time. Briefly, for the in vitro release of the encapsulated compound, 2 mL of NLC-QU were placed inside a dialysis bag (Spectra/Por^®^ CE MWCO 10000, Spectrum Laboratories, Inc; Rancho Dominguez, CA, USA). The dialysis bag was closed and placed in a dissolver containing release medium (ethyl alcohol 35%, pH 4), to maintain sink conditions. The medium was maintained at 37 °C under mechanical agitation at 75 rpm. Samples of the release medium were collected at predetermined times for 24 h and analyzed by HPLC. The free QU was used as control. Plots of cumulative amounts of drug released (%) from NLC-QU versus time (h) were constructed. After the release, kinetics were determined by adjusting the assay data to the mathematical model of zero order (Equation (1)), first order (Equation (2)), and Higuchi (Equation (2)), as follows:

C_t_ = C_0_ +Kt (1)

log C = log C_0_ − Kt/2.303 (2)

Q = K × t^1/2^ (3)

where C_t_ is the amount of drug released at time t, C is the percent of drug remaining at time t, Q is the cumulative amount of drug released in time t, C_o_ is the initial concentration of the drug and K is the model release constant.

For determination of the better mathematical model, different graphs were constructed, and linear regression analyis was realized. For the zero order model, the graph was obtained plotting the cumulative amount of drug released (%) by the time (h) of release. In the graph of the first order model, the data of logarithm of the percentage of remaining drug were plotted versus the time (h) of release. In addition, the graph of the Higuchi model was obtained relating a cumulative amount of drug released (%) and the square root of the time. The K (model release constant) was determined from the angular coefficients. 

### 2.5. In Vitro Studies: Internalization Assay, Cell Health, and Lipid Content

#### 2.5.1. Cell Culture

In the present study, J77A.1 mouse macrophage (ATTC^®^TIB-67™) was purchased from ATCC (Wesel, Germany) and used between passage numbers 8 and 30 from purchase. The cells were seeded (2.6 × 10^4^ cells/cm^2^) onto flasks in cultivation medium DMEM high glucose (Gibco, Darmstadt, Germany) completed with 10% fetal bovine serum (FBS Superior, Merck GmbH, Berlin, Germany) and 1% Penicillin/Streptomycin (10,000 U/mL, Gibco, Darmstadt, Germany). Cells were grown to 90% and passaged onto a new flask. For experiments, cells were seeded (1.5 × 10^4^ cells/ 100 µL/ well) onto bottom µclear black 96-well plates (Greiner Bio-One, Frickenhausen, Germany) with the completed cultivation medium without phenol red. J774A.1 cells were grown for 24 h until the treatment (*n* = 3 different passage numbers). 

#### 2.5.2. Internalization Assays

The J774.A1 cells were seeded in 96-well plates at an initial density of 1.10^5^ cells/well and incubated for 24 h at 37 °C with a 5% CO_2_ atmosphere. After this period, the cells were exposed to NLC-PEG-660 stearate, NLC-C18PEG900GlcNAc, and NLC-WGA (3 mg/mL of total solid content. All the formulations were prepared with 0.01% of Nile red (NR). NR diluted to the same concentrations found in the nanocarriers was used as a control for free fluorophore. After 24 h of incubation, the cells were washed 3 times and resuspended in 100 µL of PBS. For the analysis of flow cytometry, the intensity of the NR was analyzed in an Attune^®^ Acoustic Focusing Flow Cytometer (Applied Biosystems, Waltham, MA, USA). The detected material that was not a cell was removed by FSC (Forward Scatter (Applied Biosystems, Waltham, MA, USA)) x SSC (Side Scatter (Applied Biosystems, Waltham, MA, USA)) analysis. The Hoechst 33.342 (nuclei staining dye) was used at a concentration of 25 µM (Sigma-Aldrich Co., St. Louis, MO, USA). A total of 10,000 positive events for Hoechst 33.342 with a flow of 200 cells/second were analyzed using the Cytometric Attune Software V2.1 (Life Technologies, Carlsbad, CA, USA) program. Yo-Pro 1 was used to determine viable cells, at a final concentration of 0.1 μM (Invitrogen, Eugene, OR, USA). The cells were selected and classified into viable cells (negative green fluorescence = without Yo-Pro 1) and non-viable cells (high green fluorescence = Yo-Pro 1 in cellular DNA). The nanoparticle cell internalization was determined using NR, which emits orange fluorescence by cell internalization, the average orange intensity being measured only in viable cells (negative Yo-pro1). The intensity of green (Yo-Pro 1), orange (NR), and blue fluorescence (H33.342) recorded the following respective excitation/emission wavelengths: 530/30 nm, 574/26 nm (laser 488 nm), and 450/40 nm (405 nm laser). 

The J774.A1 macrophages were incubated for 24 h in 96-well plates (1.5 × 10^4^ cells/well) in a medium without the red phenol pH indicator. After this period, the cells were exposed for 24 h to the NLC PEG-660 and NLC-WGA both systems loaded with a conjugated polymer, the so-called CN-PPV (1400; 700; 350; 175; 87.5; 43.8; 21.9; 10.9 µg/mL). The use of CN-PPV allowed evaluation of the phagocytosis capacity by fluorescence intensity and by imaging of cells. For the images, a concentration of 700 µg/mL (total solids content) was used, containing 1.78 µg of CN-PPV and 25.4 ng of WGA-FITC. For FITC, the excitation and emission wavelengths 469/525 nm were used, respectively, while for CN-PPV 531/593 nm. The cells were rinsed with PBS and 100 µL of a cocktail of the nucleus marker Hoechst 33.342 (10 mg/mL) and paraformaldehyde (3.7%). After 30 min, the plate was washed with PBS at 4 °C. The fluorescence readings per well and cell were performed at the following excitation and emission wavelengths, respectively: nucleus marker: 377/447 nm; FITC: 479/520 nm and CN-PPV: 450/600 nm. The fluorescence readings and images were obtained using a Cytation 5 (BioTek, Winooski, VT, USA) and analyzed using the Gen5 software (BioTek). 

#### 2.5.3. High Content Analysis (HCA) 

A previously described multi-parameter in vitro assay was used here to study the cellular health status [28,29]. Briefly, cells were seeded (2.6 × 10^4^ cells/cm^2^) onto flasks in cultivation medium DMEM high glucose completed with 10% fetal bovine serum and 1% Penicillin/Streptomycin. Cells were grown to 90% and passaged onto a new flask. For experiments, cells were seeded (1.5 × 10^4^ cells/ 100 µL/ well) onto bottom µclear black 96-well plates with the completed cultivation medium without phenol red. J774A.1 cells were grown for 24 h until the treatment. The cells were exposed to the different NLC and control substances for 48 h. After the incubation time, cells were stained with fluorescence markers, and image analysis was performed with a cell imaging multi-mode reader Cytation 5 (BioTek Instruments GmbH, Bad Friedrichshall, Germany). Parameters such as total cell counts, increased or reduced mitochondrial activity, membrane permeability, cell area, nuclear area, number of polynucleated cells, phospholipid content, and neutral lipid content were analyzed.

##### Cell Health

For cell health, the cell count, increased or reduced mitochondrial activity, and membrane permeability as well as the vacuole area were analyzed. After the 48 h, the cells were incubated for 30 min at 37 °C with a dye cocktail including 1 mM Mito Tracker (Invitrogen™, Thermo Scientific, Hamburg, Germany), Image-iT DEAD (Invitrogen™, Thermo Scientific, Germany), and Hoechst 33342 (Invitrogen™, Thermo Scientific, Germany). After a washing step with PBS, cells were fixed with 4% paraformaldehyde in PBS for 15 min at room temperature. After the staining, image analysis was performed with Cytation 5 (BioTek Instruments GmbH, Bad Friedrichshall, Germany). All parameters are expressed as a percentage of the mean value normalized for untreated cells.

##### Lipid Content

For the phospholipid and neutral lipid content, a second assay was used. Besides the treatment with the different NLC, cells were also incubated with LipidTOX™ Red (Invitrogen™, Thermo Scientific, Germany), a fluorescence marker to detect the phospholipid accumulation in the cells. As a control for phospholipid accumulation Amiodarone hydrochloride was used. After 24 h, cells were fixed and stained with a dye cocktail containing 4% paraformaldehyde and Hoechst 33342 (Invitrogen™, Thermo Scientific, Germany) for 30 min at room temperature. Subsequently, cells were washed with PBS and incubated for an additional 30 min with the second dye cocktail containing LipidTOX™ Green (Invitrogen™, Thermo Scientific, Germany) in PBS. After the staining, image analysis was performed with Cytation 5 (BioTek Instruments GmbH, Bad Friedrichshall, Germany). All parameters are expressed as a percentage of the mean value normalized for untreated cells. The Gen5 software was programmed to automatically calculate the percentage of abnormal cells in the treated cell population in each well compared to the mean values of the untreated cells in six wells on the same plate.

### 2.6. Statistical Analysis

Statistics were calculated by a one-way ANOVA test followed by the Tukey test for means comparison and the Levene test for equal variance. The tests were performed using GraphPad Prism 7. For the HCA assay, Student *t*-tests were performed using GraphPad PRISM version 9 (GraphPad Software Inc., San Diego, CA, USA) to assess the statistical significance between two data sets with equal variance. Statistical significance was evaluated at a 95% confidence level (*p* < 0.05).

## 3. Results

### 3.1. Design of Lipid Nanocarriers: Development and Characterization

#### Particle Size, Polydispersion Index (PDI), and Zeta Potential of the Nanocarrieres Produced by High-Pressure Homogenization

The NLCs were successfully developed by HPH. To produce NLC with a modified surface, aqueous phase commercial surfactant was used in combination with 50% of the amphiphilic surfactant modified with the glycoside N-acetylglucosamine (C_18_PEG_900_GlcNAc) using the HPH technique. The NLCs with WGA on the surface were obtained by post-insertion. Table 2 shows the data for size analysis and PDI of the NLC produced with modified surfactants with and without the addition of WGA. The size of the nanocarriers remained within the same range (~150 nm) when prepared with Glc Nac in both conditions when compared to the formulation prepared only with PEG-660 stearate. The PDI also remained comparable, indicating that the systems kept within a narrow size range despite the addition of a proportion of the surfactant modified with sugar and WGA protein. The zeta potential showed that, when the surface was functionalized with WGA, the value went from approximately −28 mV (when the modified surfactant was used) to approximately −18 mV in NLC-WGA. This change in zeta potential is a strong indication that the protein has bound to the surface of the nanocarriers. These data corroborate with the results observed in our study [20].

### 3.2. Evaluation of the Presence and Activity of Lectins on the Surface of the Nanocarriers

#### 3.2.1. Determination of Hemagglutination Activity

The ability of WGA to agglutinate erythrocytes is widely described [30]. The functionalization of the NLC was confirmed by the hemagglutinating activity to determine the binding activity using the blood of rats. Briefly, the initial protein solution (1 mg/mL) was analyzed, and the hemagglutinating activity was confirmed, demonstrating the ability of the proteins used for the functionalization tests. The nanocarriers that had only the sugar on the surface (without WGA lectin) did not show hemagglutinating activity. However, the NLC containing 50% of modified surfactant and WGA showed good hemagglutinating activity. This result shows that this proportion is satisfactory for the functionalization of the nanocarriers.

#### 3.2.2. Enzyme-Linked Immunosorbent Assay (ELISA)

A validation curve was performed to validate an ELISA to quantify the WGA in the formulation. The calibration curve was linear (*y* = 0.0001x + 0.413; R² = 0.995), with a detection limit of 59.6 ng/mL and a quantification limit of 180.8 ng/mL. The amount of WGA dosed in the formulations was approximately 2000 ng/mL. The technique was able to quantify the amount of WGA present in the formulation and confirm the biological activity of the protein. The interaction of WGA with the anti-WGA antibody was successfully detected indicating that the binding site remained active after the protein binding with nanocarriers.

### 3.3. Drug Content and Release Profile

**Figure 1:**
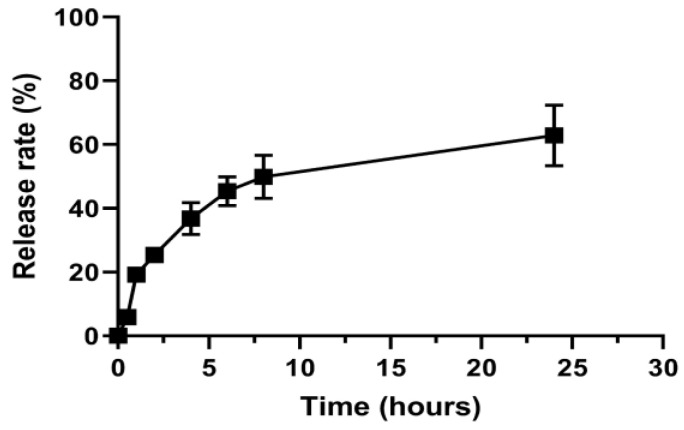
Cumulative percentage of quercetin released from NLC-QU.

The compound QU was chosen here because the compound has relatively poor water solubility, being a good model of a lipophilic drug. The drug content was determined by HPLC and showed that it was possible to encapsulate 1.5 mg/mL of QU. Figure 1 shows the in vitro release profile of quercetin from NLC-QU in ethanol: distilled water (35:75, *v*/*v*; pH 4.0) at 37 °C. Release experiments were carried out at pH 4.0 due to the low stability of quercetin in neutral and basic pH values.

The experiments were carried out in sink conditions, since the maximum concentration of QU that was reached in the release medium corresponded to 10% of its saturation concentration (1.8 mg/mL). As can be seen, the results demonstrated a sustained release of QU for 24 h, with around 63% of the drug released in this period. The free QU was used for control of the method, with 50% and 80% of the drug being detected in the release medium after 2 and 8 h, respectively. In the analysis of the QU release kinetics from NLC-QU, all times were used to construct the graphs and perform the linear regression, since, in the 24-h period, the release plateau was not observed. The evaluation showed that the data fit better in the Higuchi model (r^2^ = 0.9144), demonstrating that the release is controlled by the drug diffusion process through the nanocarrier matrix (Table 3). After confirming the Higuchi model as the most suitable to adjust the data of NLC-QU release, the release mechanism using the Koresmeyer–Peppas model and the data up to 60% of release from the initial concentration were evaluated [26,27]. In this analysis, the correlation coefficient (*r^2^*) obtained was 0.9081, and the diffusion coefficient (*n*) was 0.7, which indicates an anomalous transport (non-Fickian diffusion), in which the release mechanism is governed by the diffusion and rearrangement of the nanocarrier structure, in a swelling or similar behavior. The analysis of release kinetics is important to define how the nanocarrier interferes in the drug release.

### 3.4. Flow Cytometry

For this evaluation, nanocarriers were prepared, using the method described in Section 2.1, with the addition of a fluorescent marker in the oil phase (Nile red). The fluorescence intensity of the NR was analyzed. At the highest concentration tested (2750 µg/mL), the cells incubated with NLC-NR-WGA presented a higher fluorescence intensity of NR when compared to NLC-NR, indicating that WGA functionalization was more efficiently incorporated by cells. However, no differences were found in the NLC-NR-C18PEG900GlcNAc. Moreover, an increase in cellular volume and complexity was observed for cells exposed to all NLCs. A decrease in viable cells was observed when cells were exposed to different NLCs when compared to NR control (Table 4).

### 3.5. Internalization of the Particles Functionalized with WGA Lectin

**Figure 2:**
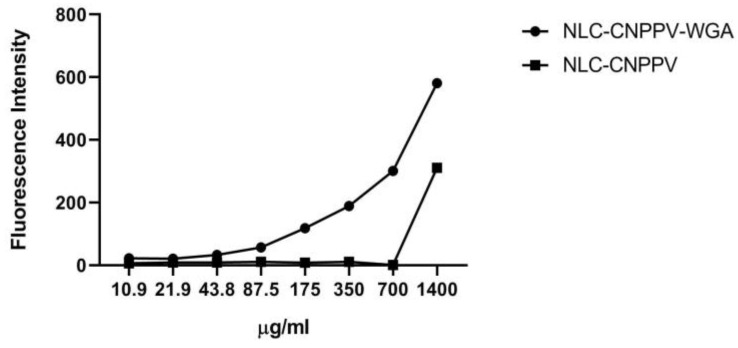
Fluorescence measurements internalized by cells after 24 h exposure to NLC-CN-PPV functionalized with the WGA protein (NLC-CN-PPV-WGA) or not (NLC-Solutol CN-PPV).

CN-PPV was used as a fluorescent marker to evaluate the phagocytosis capacity of the macrophages. The study compared the nanocarriers prepared using only PEG-660 stearate and functionalized with WGA lectin (Figure 2). The higher phagocytic activity was elucidated by the increase of the fluorescence per well. The nanocarriers functionalized with WGA showed higher phagocytic activity in a concentration-dependent manner.

**Figure 3:**
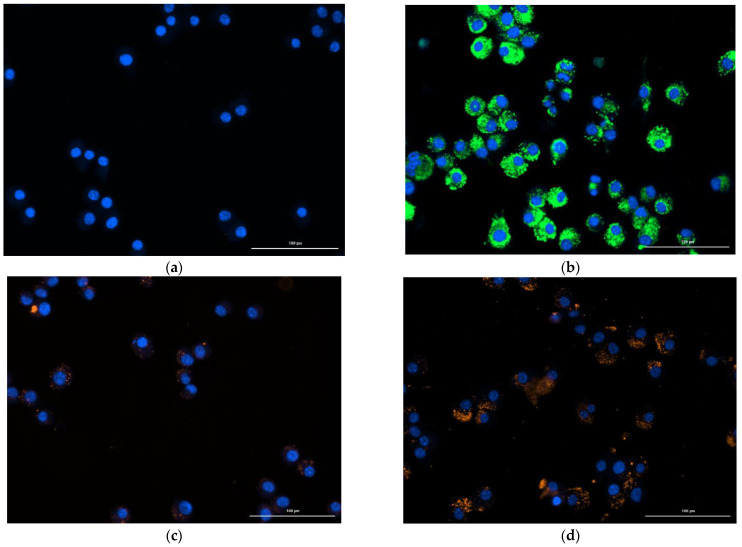
(**a**) J774.A1 control, without treatment; (**b**) J77A.A1 exposed to NLC with WGA with FITC. Ex:469; Em: 525; (**c**) J774.A1 exposed to NLC without surface functionalization with CN-PPV; (**d**) J774.A1 exposed to NLC-WGA with CN-PPV. Ex: 531; Em: 593.

Figure 3a shows the control cells, without any treatment. These cells, when analyzed with filters 469/525 nm and 531/593 nm, did not show auto-fluorescence. In Figure 3b, the cells after incubation with NLC-CN-PPV-WGA (WGA conjugated with FITC) for 24 h are displayed. After washing and fixing, it was possible to see in the green dots that the nanocarriers were internalized by the cells. In Figure 3d, it can be seen that the functionalization of the surface increased the fluorescent intensity inside the cells, compared with the NLC-CN-PPV exposure without WGA on the surface Figure 3c. When the cells were exposed to the same amount of nanocarriers, in Figure 3b, it was possible to observe a higher amount of orange signal, representing CN-PPV.

### 3.6. High Content Analysis (HCA)

The most used cell-based toxicological studies for early stages of drug development have some limitations when applied for medical nanoparticles assessment, basically because the nanomaterials can interfere with the test reagents or at the assay readout, causing false predictions [31]. To overcome those limitations, here we proposed to access the cell health status using HCA. HCA provides quantitative data on morphometric parameters and vacuolation patterns and can be combined with a variety of markers for the phenotypic and functional characterization of macrophages [32].

**Figure 4:**
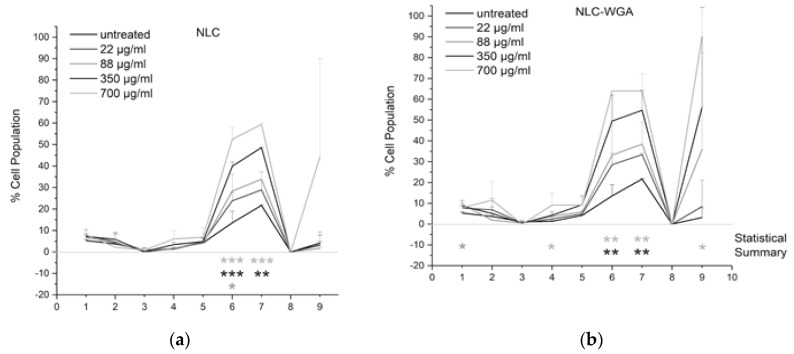
(**a**) Morphometric analysis of the macrophages exposed to blank lipid nanocarriers (NLC); (**b**) morphometric analysis of the macrophages exposed to lipid nanocarriers grafted with WGA. * *p* < 0.05; ** *p* < 0.01 and *** *p* < 0.001.

For each parameter analyzed, the percentage of the population with “abnormalities” was calculated for *n* = 3 experiments, and the numerical definitions of abnormalities are shown in Table 5. Based on the untreated cell population (*n* = 6 wells per plate), cells are considered healthy or unaffected by the NLC when <10% of the population shows abnormalities for any given parameter. The parameters of cell health, morphology, and lipid profile descriptors were classified as those that were elevated from untreated controls. ‘Elevated’ responses were defined as being greater than two standard deviations above the mean values. It is important to note that none of the NLC provoked a significant effect on the vital parameters such as mitochondrial activity and membrane permeability; on the other hand, some pronounced effects could be observed in the lipid content (Figure 4A).

The more pronounced elevated neutral lipid was observed when the cells were treated with NLC-WGA. The elevated neutral lipids are statistically significant for the NLC-WGA, being also an indication of a higher uptake that could be an effect of the WGA on the surface of the nanoparticles, that are composed of 50% with a triglyceride (Trimyristin) so an elevation in the neutral lipid was expected (Figure 4b). We also hypothesized that the elevated number and size of the vacuoles are due to the augmentation of neutral lipids inside the cells, corroborating our previous assumptions that, when the WGA is presented on the cell surface, the NP internalization is higher.

## 4. Discussion

Treating intracellular pathogens remains a pharmaceutical challenge despite all the advanced technologies. As an example, tuberculosis is still one of the greatest public health problems worldwide, despite a well-established standard of first-line treatment, there are some problems related to this regimen like adverse effects of the drugs (lack of following-up) or drug resistance. One strategy that has been recently explored is administering inhaled drugs by the pulmonary route aiming to reach higher local drug concentrations. With the use of nanotechnology, it is possible to use targeting moieties that directly or indirectly can make inhalable medicine delivery systems attractive as the drugs can reach the target site of the infection at an intracellular level [20].

Here we described a novel lipid nanosized system coated with WGA lectin aiming to increase the intracellular drug intake. The system showed an increase in the internalization of nanocarriers by macrophages when functionalized with the WGA protein in both proposed internalization tests. The strategy proposed was to take advantage of a nanostructured lipid system and its ability to entrap poorly soluble drugs plus the WGA targeting. Compared to the traditional colloidal carriers (liposomes or polymeric nanoparticles), the NLC can be produced with low costs of raw materials, providing formulations with higher physical stability and high encapsulation efficiency, with feasible industrial scale-up. Furthermore, we could confirm the ability of the NCL solid matrix to protect chemically unstable drugs and allow controlled release by using, as the lipophilic compound QU, where the best-fitted model was the Higuchi. The same kinetic model was already described [18] when solid lipid nanoparticles WGA-grafted were entrapped with rifampicin. In both cases, it is possible to assume that the release is controlled by the drug diffusion process through the nanocarrier matrix. In addition, NLC bears the advantage of being low in vivo toxicity, owing to the use of biocompatible and biodegradable materials [33].

Aiming to augment the cell–nanoparticle interaction, we design novel lipid nanocarriers coated with WGA. Several lectin-mediated drug delivery routes are proposed, such as mucoadhesion, cytoadhesion, cytoinvasion, and transcytosis [8,18,34,35,36]. The binding of lectins to the alveolar epithelium and subsequent endocytosis, as it occurs in the intestine, was already described [37]. In our previous studies [20], different lipid nanocarriers were produced and showed no cytotoxicity in the concentration range that macrophages were exposed to. Furthermore, once the nanocarriers were exposed to *M. tuberculosis*, no increase in growth was observed meaning that the products of the lipid metabolism would not be used as an energy source for the microorganism. Therefore, we could conclude that the lipid nanocarriers were feasible for further studies aiming to develop them as a carrier for drug delivery with the potential to be employed as an inhaled system. 

The results obtained strongly support the hypothesis that the WGA is grafted on the nanoparticles’ surface. The zeta potential values were approx. −28 mV when the nanocarriers were produced using the novel glycol surfactants; when WGA was added to the system, this value changed to −19 mV. A previous work confirmed the connection of the WGA to lipid nanocarriers using carbodiimide reaction after observing a change in the zeta potential (from −28 mV to approximately −17 mV), which supported us to confirm that the WGA is successfully bound to the nanocarrier’s surface [18]. Hybrid nanocarriers composed of lipids and polymers (LPN) were produced by incubating synthetic WGA-1,2-dioleoyl-sn-glycerol-3-phosphoethanolamine with LPNs, which had been formed using nanoprecipitation (200 nm) aiming to increase intestinal bio adhesion promoting an increase in the concentration of drugs inside the cells. Functionalization with WGA facilitated the binding of the nanocarriers to the intestinal epithelium, further increasing the internalization of the encapsulated drug in vivo and in vitro. This effect was attributed to receptor-mediated endocytosis when recognizing WGA on the nanocarrier surface [38]. 

Lipid nanoparticles were produced by the high-pressure homogenization method [39], and the WGA was covalently bound to lipid nanoparticles by the glutaraldehyde method and the carriers obtained (~160 nm) enhanced the binding and uptake of the nanoparticles with Caco-2 (gut) cells. They assumed that the enhancement of the cell-nanoparticle association effect was caused by an energy-dependent mechanism plus the sugar-specific interactions. The nanoparticle-WGA binding methods described before are made in two main steps, covalent coupling, and physical adsorption. The most used coupling methods are glutaraldehyde and carbodiimide coupling, and these processes normally are time-consuming due to the ultracentrifugation, incubation, and dialysis steps (among others). The direct grafting of WGA with lipid nanoparticles is described in the literature as a difficult process due to the limited number of functional groups on the surface of nanoparticles [39]. Here we present a new method, wherein the novel surfactants used for lipid nanoparticle production are already modified with the specific sugar moiety that allows WGA to bind specifically.

The WGA was successfully grafted to the surface of the nanoparticles, and it was possible to confirm by the ELISA assay that the lectin remained active after the binding to the surfactant’s glycoconjugates. The WGA lectin has an already described potential to improve drug delivery by using WGA-grafted nanoparticles to facilitate adhesion and uptake of therapeutics and enhance therapeutic efficacy [40]. Here, the capacity of uptake of NLC-WGA by macrophages was analyzed using the fluorescent marker NR, incorporated into the nanocarriers, and we could conclude from the flow cytometry data that, when the WGA was grafted on the nanoparticles surface, a higher amount of NR was detected. The functionalization of NLC with WGA contributes to a greater uptake of nanoparticles by macrophages, and we could confirm this trend when the nanocarriers were entrapped with CN-PPV for the HCA.

The HCA was designed to be an early non-clinical screening tool to predict the safety of candidate medicines [29]; therefore, a multi-parameter assay approach was used here to generate a more detailed characterization of how the nanoparticles impact macrophage responses. It was possible to access the cellular health status plus a higher NLC-WGA uptake shown by the cellular neutral lipid augmentation when compared to the NLC without lectin. The novel lipid systems WGA grafted that are proposed here can be explored for delivering novel or repurposed anti-infective agents, targeting intracellular sites of infection, thereby helping to increase the therapeutic index of a drug in intracellular niches, while avoiding problems associated with administering high doses of anti-infectives systemically. Another promising approach would be to apply the system developed here for the prophylaxis and treatment of SARS-CoV-2 infection.

## 5. Conclusions

A lipid nanocarrier with the surface-functionalized with the WGA protein was successfully produced and characterized in terms of size, polydipersion index and zeta potential. The system was able to incorporate a high amount (1.5 mg/mL) of the hydrophobic model compound (quercetin), and it was released in a controlled manner via a non-Fickian diffusion-based release mechanism. The nanocarriers surface functionalization with the WGA protein increased expressively the phagocytosis by macrophages in two different internalization assays. The phagocytosis was WGA-nanocarrier concentration-dependent. The developed system demonstrates a promising capacity to deliver intracellular drugs, being encouraged to be further studied for the delivery of anti-infectives to treat intracellular pathogens. Particularly considering the treatment of diseases such as tuberculosis, the formulation developed is a promising candidate for pulmonary administration.

## Figures and Tables

**Table 1:** Composition of the nanocarriers NLC prepared with the association of the PEG-660 stearate and modified surfactant (C_18_PEG_900_GlcNAc).

Excipients	Amount for 10 mL (mg)	% of Each Compound in Relation to Total Solids
Egg lecithin E80	100.0 mg	18.1
Trimyristine	280.0 mg	50.8
Castor oil	120.0 mg	21.7
PEG-660 stearate	25.0 mg	4.55
C_18_PEG_900_GlcNAc	25.0 mg	4.55

**Table 2:** Results of size, PDI, and zeta potential of the formulations produced by HPH when 50% of modified surfactants were added to the system with and without the addition of the WGA protein measured by DLS.

Nanocarrier	Size (nm)	PDI	Zeta Potential (mV)
NLC ^1^	159.8 ± 3.42	0.144 ± 0.01	−23.8 ± 1.11
NLC-Glc Nac	157.3 ± 1.63	0.111 ± 0.01	−28.5 ± 0.08
NLC-WGA	157.6 ± 1.10	0.095 ± 0.01	−19.0 ± 0.96
NLC-QU	121.9 ± 0.05	0.170 ± 0.022	−13.5 ± 0.702

^1^ Blank-NLC produced with 100% of PEG-660 stearate as an aqueous phase surfactant.

**Table 3:** Kinetics parameters obtained from the nanoparticles release profile.

	Zero-Order	First Order	Higuchi
Equation	Y = 2.2919x + 17.64	Y = −0.0166x + 1.9147	Y = 13.779x + 4.3262
Correlation coefficient (*r^2^*)	0.6828	0.8029	0.9144
Constant release (*k*)	2.2919	0.0382	13.779

**Table 4:** Results of fluorescence intensity inside cells after 24 h of exposure to the proposed treatments. The data were expressed as mean ± standard deviation. Different letters on the same column indicate significant differences based on the Kruskal–Wallis non-parametric test with a significance limit of 0.01.

Treatment	Cell Volume—FSC (×10^6^)	Cellular Complexity (×10^6^)	Viable Cells (%)	Cellular Internalization of the NR
NR	1.62 ± 0.06 ^b^	2.22 ± 0.37 ^b^	92.9 ± 1.8 ^b^	3468 ± 591 ^c^
NLC-NR	2.10 ± 0.13 ^a^	2.73 ± 0.15 ^a^	81.6 ± 3.9 ^a^	16021 ± 5805 ^bc^
NLC-NR-C18PEG900GlcNAc	2.04 ± 0.11 ^ab^	2.67 ± 0.14 ^a^	84.3 ± 3.4 ^a^	31260 ± 8647 ^ab^
NLC-NR-WGA	2.10 ± 0.15 ^a^	2.92 ± 0.12 ^a^	82.7 ± 4.5 ^a^	50628 ± 14459 ^a^

**Table 5:** Values above or below the ones stated for each parameter were considered abnormal.

No.	Parameter		No.	Parameter	
1	Abnormal nuclear area	<74 µm^2^ or >194 µm^2^	6	Elevated vacuole number per cell	>1 with >0.9 µm^2^
2	Elevated mitochondrial activity	FI > 8914 a.u.	7	Elevated vacuole area per cell	>4 µm^2^
3	Reduced mitochondrial activity	FI < 3098 a.u.	8	Elevated phospholipid content	FI > 91 a.u.
4	Elevated membrane permeability	FI > 7397 a.u.	9	Elevated neutral lipid content	FI > 6986 a.u.
5	Elevated cellular area	> 642 µm^2^			

## Data Availability

The raw data generated from this study are available from the corresponding author G.H. on request.
